# Assessment of Brain-Derived Neurotrophic Factor on Retinal Structure and Visual Function in Rodent Models of Optic Nerve Crush

**DOI:** 10.3390/ph17060798

**Published:** 2024-06-18

**Authors:** Takazumi Taniguchi, Najam A. Sharif, Takashi Ota, Rafal A. Farjo, Rebecca Rausch

**Affiliations:** 1 Ophthalmology Innovation Center, Santen Pharmaceutical Co., Ltd., Nara 630-0101, Japan; 2 Ophthalmology Innovation Center, Santen Inc., Emeryville, CA 94608, USA; 3 Institute of Ophthalmology, University College London, London WC1E 6BT, UK; 4 Imperial College of Science and Technology, St. Mary’s Campus, London SW7 2AZ, UK; 5 Eye-APC Duke-NUS Medical School, Singapore 169857, Singapore; 6 Department of Pharmacy Sciences, Creighton University, Omaha, NE 68178, USA; 7 Department of Pharmaceutical Sciences, College of Pharmacy and Health Sciences, Texas Southern University, Houston, TX 77004, USA; 8 Department of Pharmacology and Neuroscience, University of North Texas Health Sciences Center, Fort Worth, TX 76107, USA; 9 Singapore Eye Research Institute, Singapore 169856, Singapore; 10 Global Research and Development, Nanoscope Therapeutics Inc., Dallas, TX 75207, USA; 11 EyeCRO LLC, Oklahoma City, OK 73105, USA

**Keywords:** brain-derived neurotrophic factor, optic nerve crush, retinal ganglion cells, glaucoma, neuroprotection

## Abstract

The effects of brain-derived neurotrophic factor (BDNF) on retinal ganglion cell (RGC) survival and visual function were assessed in rat and mouse models of optic nerve (ON) crush. ONs were crushed on Day 1, followed by intravitreal injections of a vehicle or BDNF on Days 1 and 8. The spatial frequency threshold was measured using optokinetic tracking on Days 7 and 14. On Day 15, ganglion cell complex (GCC) thickness was quantified using optical coherence tomography. Furthermore, all eyes were enucleated for immunohistochemical analysis of the surviving RGC somas and axons. BDNF significantly reduced the RGC soma in mice and increased GCC thickness in intact eyes, with apparent axonal swelling in both species. It displayed significantly greater RGC soma survival in eyes with ON injury, with moderately thicker axonal bundles in both species and a thicker GCC in rats. Visual function was significantly reduced in all ON-crushed animals, regardless of BDNF treatment. Thus, we obtained a comprehensive analysis of the structural and functional impact of BDNF in intact and ON-crushed eyes in two rodent models. Our results provide a foundation for further BDNF evaluation and the design of preclinical studies on neuroprotectants using BDNF as a reference positive control.

## 1. Introduction

Glaucoma, a major optic neuropathy, is the leading cause of irreversible vision loss and blindness worldwide [1,2]. This complex neurodegenerative disease affects the retinal ganglion cell (RGC) somas and axonal projections that comprise the optic nerve [1,3]. Glaucomatous optic neuropathy (GON) is characterized by damage to the optic nerve head, with progressive cupping and distinctive progressive changes in the visual field. Elevated intraocular pressure (IOP) is a major risk factor for the development and progression of glaucoma, and lowering the IOP pharmaceutically or surgically is the only known treatment option for this disease [1,3]. However, further visual field deterioration continues despite good IOP management in some cases. This suggests that the presence of IOP-independent mechanisms, such as neurotrophic factor deprivation, oxidative stress, ocular blood flow disturbance, and inflammation, contributes to the development and progression of glaucoma [3,4,5,6]. Therefore, new treatment options that directly protect RGCs and the optic nerve in an IOP-independent manner are needed to prevent blindness. Many neuroprotective strategies using small-molecule drugs in addition to cell and gene therapies with various modes of action have been proposed. However, there remain no approved treatment options to date [3,4,5,6].

Animal models that mirror or closely resemble human disease mechanisms are critical to elucidating disease etiology and molecular pathobiology. These models enable the progression of new therapies into clinical development [7]. To obtain meaningful preclinical data on candidate neuroprotective agents for glaucoma during drug discovery and development, the establishment of clinically relevant animal models with non-IOP-related factors associated with glaucomatous damage to the RGCs and/or the optic nerve is critical. One of the hypothesized pathogeneses of GON is the local damage of lamina cribrosa, disrupting axoplasmic transport within the optic nerve fibers [1,3]. Therefore, animal models in which forceps or a surgical clip are used to crush the optic nerve in a controlled manner, thereby causing specific RGC soma and optic nerve degeneration in an IOP-independent manner, have been extensively used to evaluate candidate neuroprotective agents in preclinical settings in glaucoma research. Additionally, these models have been used to study the pathophysiological events involved in RGC damage caused by the loss of retrograde transport of trophic factors [8,9,10,11,12,13,14,15]. Thus, optic nerve crush injuries in rats and mice have become popular animal models with several advantages. Optic nerve injury caused by mechanical crush in these species is relatively easy to establish and highly reproducible. In addition, rat and mouse eyes have several similarities to the human eye, although the non-human primate eye has the closest anatomical structure to that of humans [13,14]. Furthermore, because rodents are much less expensive to purchase and maintain than non-human primates, larger numbers can be used to obtain statistically meaningful data [13]. Many potential agents with neuroprotective effects have been identified from various studies using these optic nerve injury models [8,9,10,11,15].

Neurotrophic factors, particularly brain-derived neurotrophic factor (BDNF), exert a robust neuroprotective effect on RGC somas histologically analyzed in rodent optic nerve crush models [10,15,16,17]. This factor confers neuroprotective effects directly via the Tropomyosin receptor kinase B (TrkB) expressed in RGCs and/or indirectly via the TrkB expressed in glial cells. BDNF is locally produced by RGCs and other retinal cells, but the retrograde transport of BDNF from the brain to the retina is crucial [18]. In addition to the interruption of this retrograde transport in the early stages of glaucoma, lower levels of BDNF were detected in the serum and ocular fluid of patients with glaucoma compared to people without the disease, supporting the theory that neurotrophic deprivation is a potential mechanism of GON [18]. Although the precise factors that contribute to glaucoma are still debated, the neurotrophin deprivation theory represents one of the most predominant proposed contributors to this disease [4,5,6]. 

BDNF could be a useful agent for various purposes, such as elucidating the mechanism underlying RGC damage following optic nerve crush. In addition, it can be used as a comparator/reference active compound during the development of neuroprotective candidates. On the other hand, there is a lack of assessment of BDNF on retinal thickness using clinically useful optical coherence tomography (OCT) and on visual function measurements. These are important indices for evaluating the structure and function of candidate drugs in optic nerve crush models. Furthermore, although an understanding of the impact of BDNF in intact eyes is needed to correctly interpret the effects of this trophic factor in crushed eyes, these parameters have not yet been fully investigated.

**Figure 1:**
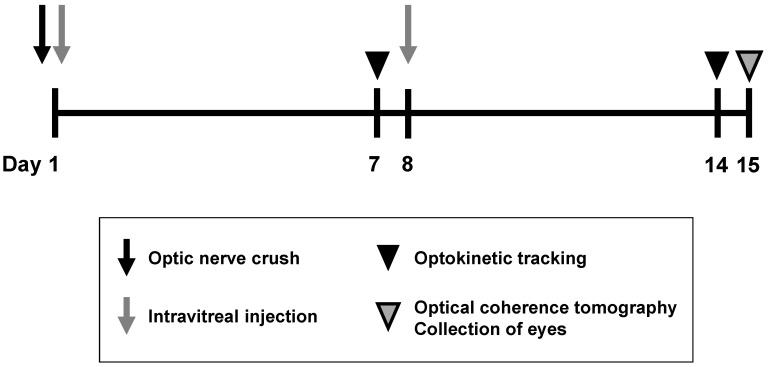
An outline of optic nerve crush experiments in rats and mice. The optic nerve was crushed just before the intravitreal injection of the vehicle, or BDNF, on Day 1. A second intravitreal injection was performed on Day 8. Optokinetic tracking was performed to assess visual function on Days 7 and 14. Optical coherence tomography was performed to quantify the ganglion cell complex (GCC) thickness on Day 15, followed by animal euthanasia, after which eyeballs were enucleated for flatmount immunohistochemistry to label retinal ganglion cells (RGCs) with RBPMS and TUJ1.

In this study, we simultaneously assessed the effects of intravitreal injection of BDNF on RGC soma survival, retinal thickness, and visual function in naïve and crushed optic nerve eyes of rats and mice, as illustrated in Figure 1. Additionally, data were compared between the two species.

## 2. Results

### 2.1. Retinal Ganglion Cell Survival

**Figure 2:**
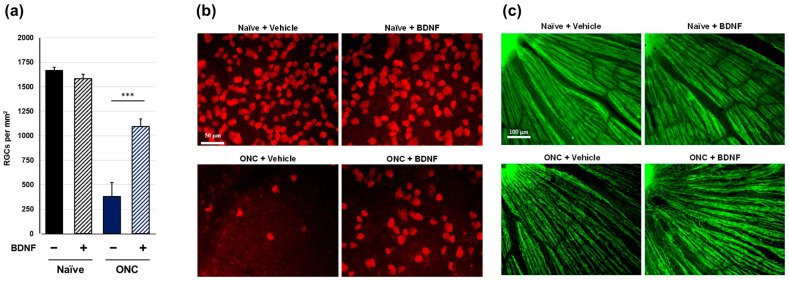
Effects of BDNF on retinal ganglion cell survival in rats. RGC soma count (**a**), as measured in uncrushed and crushed eyes administered a vehicle or BDNF injection. Vehicle, closed column; BDNF, diagonal column. Each value represents the mean ± standard error of the mean (SEM) for n = 8 (**a**). *** *p* < 0.001, as determined using Student’s *t*-test. Representative images from retinal flatmounts stained with anti-RBPMS to label surviving RGCs in peripheral regions (**b**) and with anti-TUJ1 to label RGC axons close to the optic nerve head (**c**) in rats. No difference in RGC soma count was observed between the uncrushed eyes, regardless of treatment. Crushed eyes receiving BDNF displayed significantly greater RGC survival compared to those injected with the vehicle. Axonal density and integrity were decreased in the crushed eyes. However, axon bundles appeared moderately thicker in BDNF-injected eyes compared to vehicle-injected controls. Note: The bright spot in the top left represents the optic nerve head (**c**).

Rat retinal flatmounts were immunolabeled with RNA-binding protein with multiple splicing (RBPMS) antibodies to visualize RGC somas following terminal collection two weeks post-crush. Optic nerve crush resulted in a significant loss of RGC somas in the vehicle-treated group (*p* < 0.001, Aspin-Welch *t*-test). No statistically significant difference in RGC soma survival was observed between uncrushed vehicle-treated eyes and uncrushed BDNF-treated eyes, although there was a slight trend toward a reduction in the BDNF group. Crushed eyes injected with BDNF exhibited significantly greater RGC survival than the vehicle-injected controls (Figure 2a,b). All retinas were co-labeled with beta-tubulin III (anti-TUJ1), which labels the RGC axon in addition to the soma, to qualitatively assess RGC axon degeneration proximal to the site of the optic nerve crush injury. Uncrushed nerves showed typical RGC axon integrity regardless of BDNF injection. However, crushed nerves treated with the vehicle displayed characteristic signs of axonal degeneration, such as thinning, beading, and fragmentation. The axon bundles were moderately thicker in eyes administered with BDNF than in crushed eyes receiving the vehicle injection (Figure 2c). These findings directly correlate with the significant protection observed in RGC soma counts. 

**Figure 3:**
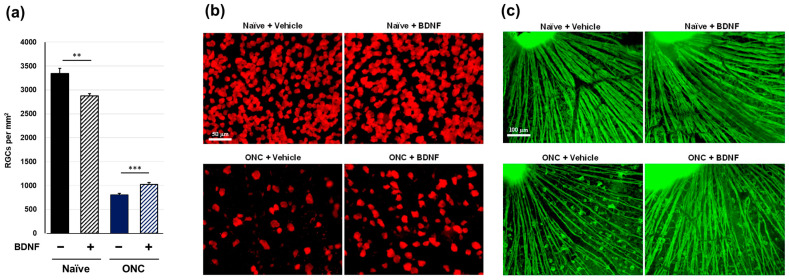
Effects of BDNF on retinal ganglion cell survival in mice. RGC soma number, as measured in uncrushed and crushed eyes administered a vehicle or BDNF injection. Vehicle, closed column; BDNF, diagonal column. Each value represents the mean ± SEM for n = 8 (**a**). ** *p* < 0.01, *** *p* < 0.001, as determined using Student’s *t*-test. Representative images from retinal flatmounts stained with anti-RBPMS to label surviving RGCs in peripheral regions (**b**) and with anti-TUJ1 to label RGC axons close to the optic nerve head (**c**) in mice. BDNF slightly decreased RGC soma survival in uncrushed eyes compared to vehicle-injected eyes. Crushed eyes receiving BDNF displayed greater RGC survival compared to those injected with the vehicle. Axonal density and integrity were decreased in crushed eyes. However, axon bundles appeared thicker in eyes administered BDNF compared to vehicle-injected controls. Note: The bright spot in the top left represents the optic nerve head (**c**).

Optic nerve crush significantly induced the loss of RGC soma in the vehicle-treated group in mouse experiments (*p* < 0.001, Aspin-Welch *t*-test). The RGC soma number was significantly reduced in BDNF-injected, uncrushed eyes. In crushed eyes, the protective effect of BDNF was small but exerted statistically significant RGC soma protection compared to vehicle-injected controls (Figure 3a,b). Uncrushed nerves labeled with anti-TUJ1 corresponded with typical RGC axon integrity, regardless of BDNF injection. However, crushed nerves with vehicle injection displayed signs of axonal degeneration. In the group receiving BDNF, the axon bundles were moderately thicker than those in the group of crushed optic nerves that received the vehicle injection (Figure 3c). These mouse-based findings of immunostaining with anti-TUJ1 were similar to those observed in rat experiments.

### 2.2. Ganglion Cell Complex Thickness

**Figure 4:**
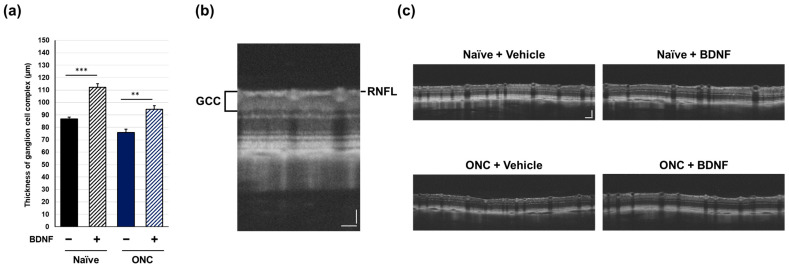
Effects of BDNF on ganglion cell complex (GCC) thickness in rats. GCC thickness (**a**), as measured in uncrushed and crushed eyes administered a vehicle or BDNF injection. Vehicle, closed column; BDNF, diagonal column. Each value represents the mean ± standard error of the mean (SEM) for n = 4–8 (**a**). ** *p* < 0.01, *** *p* < 0.001, as determined using Student’s *t*-test. Representative peripapillary circle scans from optical coherence tomography measurements in rats (**b**). The GCC thickness, which consists of the retinal nerve fiber layer (RNFL), ganglion cell layer (GCL), and inner plexiform layer (IPL), was measured. ONC reduced GCC thickness in vehicle-injected eyes. Increased thickness was evident in the RNFL of BDNF-injected eyes without optic nerve crush. A slight RNFL increase was also qualitatively observed in BDNF-injected crushed eyes (**c**). Scale bar = 50 mm.

In vivo OCT was performed immediately prior to euthanasia to examine the effect of BDNF administration after optic nerve crush on the retinal structure in rats. The ganglion cell complex (GCC), measured from the retinal nerve fiber layer (RNFL) to the inner plexiform layer and comprising RGC somas, dendrites, and axons, was quantified using circular scans acquired from the peripapillary region of the retina. Optic nerve crush caused a significant reduction in GCC thickness in the vehicle-treated group (*p* < 0.01, Student’s *t*-test). Administration of BDNF significantly increased GCC thickness in uncrushed eyes, likely due to axonal swelling in the RNFL, though this was not directly confirmed (Figure 4). This result was less apparent in crushed eyes, although GCC thickness was significantly greater in the BDNF injection group than in the vehicle group (Figure 4).

**Figure 5:**
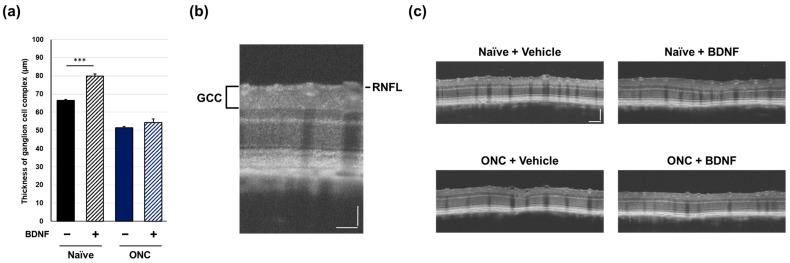
Effects of BDNF on ganglion cell complex (GCC) thickness in mice. GCC thickness (**a**), as measured in uncrushed and crushed eyes administered a vehicle or BDNF injection. Vehicle, closed column; BDNF, diagonal column. Each value represents the mean ± SEM for n = 7–8 (**a**). *** *p* < 0.001, as determined using Student’s *t*-test. Representative peripapillary circle scans from optical coherence tomography measurements in mice (**b**). The GCC thickness was measured. ONC reduced GCC thickness in vehicle-injected eyes. Increased thickness was only evident in the RNFL of uncrushed eyes administered BDNF (**c**). Scale bar = 100 mm.

In the mouse experiments, optic nerve crush caused a statistically significant decrease in GCC thickness in the vehicle-treated group (*p* < 0.001, Student’s *t*-test). Administration of BDNF significantly increased the GCC thickness in uncrushed eyes, similar to that observed in the rat experiments (Figure 5). In the crushed eyes, GCC thickness in the BDNF injection group was slightly thicker than that in the vehicle-injected group, but the difference was not statistically significant (Figure 5).

### 2.3. Optokinetic Tracking

**Figure 6:**
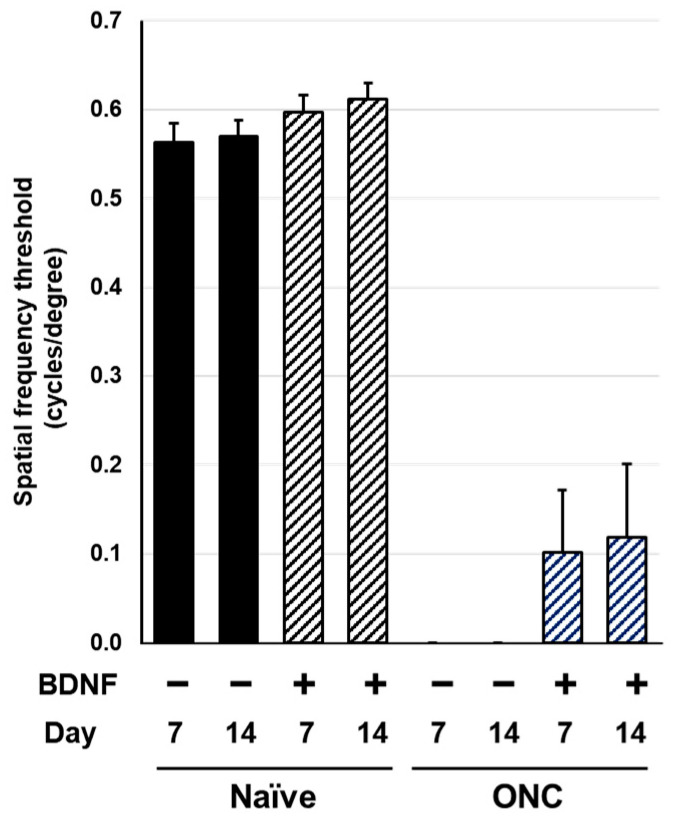
Effects of BDNF on optokinetic tracking in rats. Spatial frequency threshold, as measured in uncrushed and crushed eyes administered a vehicle or BDNF injection. Vehicle, closed column; BDNF, diagonal column. Each value represents the mean ± standard error of the mean (SEM) for n = 8–10.

**Figure 7:**
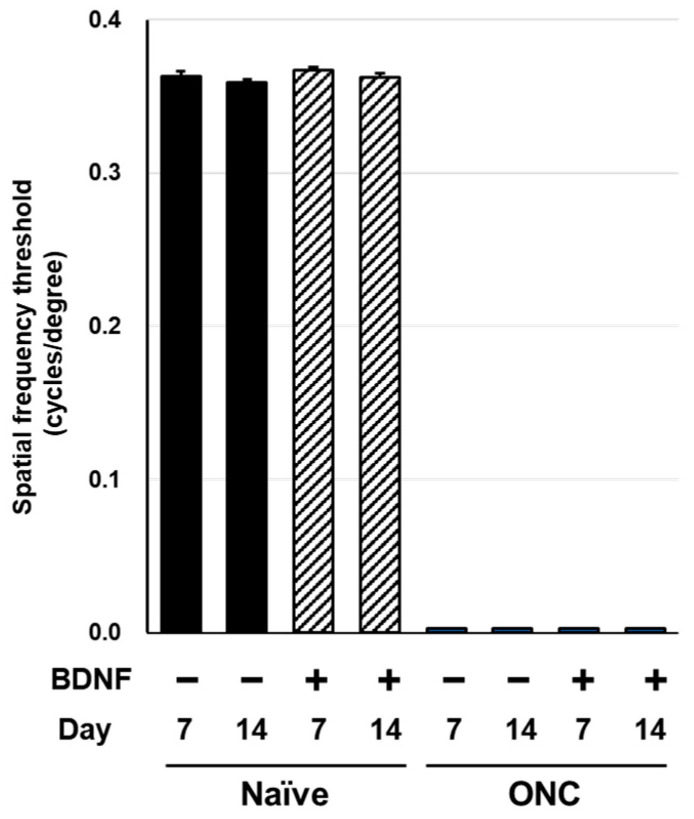
Effects of BDNF on optokinetic tracking in mice. Spatial frequency threshold, as measured in uncrushed and crushed eyes administered a vehicle or BDNF injection. Vehicle, closed column; BDNF, diagonal column. Each value represents the mean ± SEM for n = 5–8.

Spatial frequency thresholds (SFT) were analyzed on Days 7 and 14 in the rats and mice to assess visual function following an optic nerve crush injury and BDNF treatment. There was no significant difference in SFT between the uncrushed groups at either time point for either species (Figure 6 and Figure 7). A significant reduction in SFT was observed in animals with crushed optic nerves, regardless of BDNF treatment, although there was a small trend of functional recovery in the group that received BDNF in rats (Figure 6 and Figure 7).

## 3. Discussion

We simultaneously assessed the effects of intravitreally administered BDNF on RGC survival and visual function in rat and mouse eyes with intact and crushed optic nerves, providing neuroprotective effect profiles for this trophic factor in a relevant disease model. The key findings of the present study are as follows: (1) RGC soma survival is a robust index for detecting the neuroprotective effects of BDNF in the optic nerve crush injury model of GON; (2) BDNF administration causes RGC soma loss (only in mice) and increased GCC thickness in intact eyes (both species), indicating that the evaluation of BDNF in uncrushed eyes is required to properly assess the effects of BDNF in crushed eyes; and (3) the neuroprotective effects of BDNF were similar between rats and mice, with a few exceptions. 

Consistent with previous studies [15,16,17], intravitreal injection of BDNF afforded some protection of the RGC soma from optic nerve injury in rats and mice (Figure 2a and Figure 3a). The two-time injection of BDNF (10 µg/eye in rats and 2 µg/eye in mice) demonstrated that its neuroprotective efficacy was stronger in rats than in mice. In both species, the partial protective effects of BDNF on RGC death may be due to the relatively short half-life of this trophic factor [18]. On the other hand, BDNF significantly induced RGC soma loss in uncrushed mouse eyes (Figure 3a). Thus, a lower BDNF dose may exert stronger neuroprotective effects in mice. BDNF binds to two receptors (the p75 neurotrophin receptor and the TrkB receptor), and stimulation of the p75 receptor by BDNF activates cell death signaling [19,20]. The mechanism underlying the RGC soma loss observed in the intact eyes of mice is unclear. However, stimulation of the p75 receptor may be a contributing factor. 

OCT has been widely used in clinical settings to analyze retinal structures and help assess the diagnosis and progression of GON [21]. Therefore, OCT analysis in animal models is valuable for translating pre-clinical data to human subjects. Intriguingly, the intravitreal injection of BDNF increased GCC thickness in the intact eyes of both rats and mice (Figure 4a and Figure 5a). Although the causative mechanism of this potentially negative impact of BDNF on the retina is unclear, this is likely due to axonal swelling in the RNFL (Figure 4c and Figure 5c). However, this is not unprecedented, as a previous study also indicated that BDNF induces axonal swelling in the injured optic nerve following a lens injury [22]. In rats, GCC thickness in BDNF-injected eyes with crushed optic nerves was greater than that in the corresponding vehicle control group (Figure 4a). This effect seems to be reflected in the presumed direct axonal swelling effect of BDNF, which remains unclear.

Optic nerve crush completely ablated visual function, as assessed through SFT in rats and mice (Figure 6 and Figure 7). The intraretinal axons labeled with the anti-TUJ1 antibody (Figure 2c and Figure 3c) were not completely damaged, suggesting that this functional index of the SFT is more sensitive than the structural indices assessed in this study. Under the current experimental conditions, BDNF did not provide significant functional protection in either rodent species (Figure 6 and Figure 7). We crushed the optic nerve using self-closing forceps to induce mechanical injury. It is important to note that the level of optic nerve damage can vary depending on the crush technique, such as the magnitude of the applied crush force and the duration of the crush injury using forceps. Although our technique produced consistent and reproducible damage, the extent of the injury may have been too severe to allow for any positive effects of BDNF on the visual function of the animals. Interestingly, uncrushed eyes injected with BDNF did not exhibit changes to visual function (Figure 6 and Figure 7), despite GCC swelling in both species and the loss of RGC somas observed in mice. The reason behind the apparent lack of correlation between functional and structural indexes remains unclear, but intrinsic complementary mechanisms might have been activated in these uncrushed eyes. 

Our findings suggest that RGC soma survival is a more robust index for detecting the protective effects of BDNF in rodent optic nerve crush injury models compared to other structural and functional indices. However, the latter may be altered under different experimental conditions, such as the degree of optic nerve injury, administered BDNF dose, frequency of BDNF injections, and timing of evaluation following optic nerve crush. Further, extensive studies are required to confirm these hypotheses. To the best of our knowledge, only a few studies have investigated the effects of BDNF on intact rodent eyes (e.g., the effect on the TrkB receptor and GFAP expressions) [23,24]. The present study revealed the effects of BDNF on RGC soma survival, GCC thickness, and visual function in intact rodent eyes.

Overall, there were data similarities between rats and mice in the present study. Therefore, we could use either species for optic nerve crush experiments, depending on the purpose. Rats may be preferable in studies focusing on the pharmacokinetics and tolerability of candidate neuroprotective drugs, owing to their larger eyeballs, whereas the greater availability of mice makes them the ideal species for studies using genetically modified animals. Furthermore, the surgical procedure differs slightly between the two species. Rats require the use of larger and stronger self-closing forceps, more time spent exposing the nerve, and additional pain relief post-surgery. While nerve exposure is performed more quickly in mice, there is a greater chance of unintentional blood vessel laceration inhibiting proper visualization for a successful crush. Mice tend to tolerate the procedure better than rats, requiring less pain reliving intervention post-surgery. If properly crushed, both species produce highly reproducible results.

In summary, we obtained fundamental data on the effects of intravitreally injected BDNF in intact eyes and those with crushed optic nerves in rats and mice. As for the comparative standpoint between intact and crushed eyes, the impacts of BDNF were different depending on the indexes, as described above. A series of these baseline data will be useful to further analyze the effects of BDNF and its relevant receptor agonists, such as TrkB receptor agonists, on optic nerve injury in rodents. 

## 4. Methods and Materials

### 4.1. Animals

Female Brown Norway rats (10–13 weeks old) were purchased from Charles River Laboratories. Female C57Bl/6J mice (6–9 weeks old) were purchased from Jackson Laboratories. All animal studies were reviewed and approved by the Institutional Animal Care and Use Committee of EyeCRO (Ethics number #2021-07-16-001), and all procedures adhered to the ARVO Statement for the Use of Animals in Ophthalmic and Vision Research, state guidelines, and local regulations. Animals were housed in groups of 3–5 in cages and kept on ventilated shelves under standard animal care conditions (humidity: 30–70%; temperature: 20–26 °C; 12 h light/dark cycle). In addition, they had ad libitum access to water and food (LabDiet PicoLab Rodent Diet 20; #5053). 

### 4.2. Anesthesia

The animals were sedated with ketamine (rats, 60 mg/kg; mice, 85 mg/kg) and xylazine (rats, 9 mg/kg; mice, 14 mg/kg), which were administered intraperitoneally. Sedation was verified based on the lack of response to a toe pinch before any invasive procedure. Breathing and heart rate were monitored throughout the procedure until consciousness was regained.

### 4.3. Optic Nerve Crush

A small incision was made in the temporal conjunctiva of fully sedated animals, taking care to avoid the underlying musculature and vasculature. Forceps were then used to retract the conjunctiva to expose the posterior globe and allow access to and visualization of the optic nerve. To proceed with the optic nerve crush surgery, self-closing forceps were used to grasp and pinch the optic nerve approximately 1–3 mm from the globe, and the pressure was maintained for 5 s (mice) or 25 s (rats). The forceps were then removed, and a local anesthetic (proparacaine) and antibiotic ointment (gentamycin) were applied to the exposed area. Ketofen (mice and rats: 5 mg/kg) and Buprenorphine (rats only: 0.05 mg/kg) were subcutaneously injected immediately following the procedure and again at 24 and 48 h post-procedure to provide pain relief. The animals were monitored daily for signs of infection, bleeding, or loss of eye motor control. 

### 4.4. Intravitreal Injection

The animals were first sedated and dilated with Cyclomydril (cyclopentolate/phenylephrine). A pilot hole was created in the pars plana using a 30-gauge needle. A total volume of 5 µL/eye (rats) or 1 µL/eye (mice) was injected into the vitreous using a Hamilton syringe and a blunt tip 33-gauge needle and gently dispensed over 5–10 s. Care was taken to avoid damaging the lens and retina. A topical antibiotic ointment was applied to the eye following the injection. If the optic nerve crush and intravitreal injections were performed on the same day, the injections were administered immediately following the optic nerve crush. 

### 4.5. Growth Factor Formulation

Animal-free BDNF (#AF-450-02) was purchased from PeproTech Inc. (Cranbury, NJ, USA). BDNF was prepared at 2 μg/μL in 1% BSA-PBS, sterile-filtered, and stored at 4 °C. The formulations were stored on ice during dosing. The BDNF dose was determined based on prior in-house validation studies at EyeCRO.

### 4.6. Experimental Design

The optic nerve was crushed just before the intravitreal injection of the vehicle or BDNF. A second injection of the vehicle or BDNF was administered seven days after the first intravitreal injection. This injection timing was chosen based upon preliminary experiments to find the frequency that limited the potential for ocular damage caused by multiple injections. Next, optokinetic tracking was performed to measure the spatial frequency threshold to assess visual function six and thirteen days after the optic nerve crush. Fourteen days after the optic nerve crush, optical coherence tomography was performed to quantify the retinal thickness of the GCC. The animals were euthanized immediately thereafter, and the eyeballs were enucleated for flatmount immunohistochemistry to label RGCs with RBPMS and TUJ1. The time points for immunohistological assessment of RGC soma survival, spatial frequency threshold evaluation, and OCT measurements were determined in preliminary studies. The experimental design is illustrated in Figure 1.

### 4.7. Retinal Flatmount Immunohistochemistry and RGC Quantification

The enucleated eyes were fixed in 4% paraformaldehyde for 2 h at 20–26 °C before retinal dissection. Free-floating retinas were double-labeled in a 48-well plate with anti-RBPMS (GeneTex, Irvine, CA, USA, Cat #GTX118619, Lot #44181; 1:500 dilution) and anti-TUJ1 (BioLegend, San Diego, CA, USA, Cat #801202, Lot #B249869; 1:1000 dilution) primary antibodies for 48 h in PBS containing 0.3% Triton-X. They were subsequently incubated in the corresponding secondary antibodies (donkey anti-rabbit 555, Thermo Fisher Scientific, Waltham, MA, USA, Cat#A31572, 1:1000 dilution, and donkey anti-mouse 488, Thermo Fisher Scientific, Cat#A21202, 1:1000 dilution) for 24 h in PBS and flatmounted onto a microscope slide. Eight evenly spaced 40× magnified images were captured from the periphery of each retinal flatmount using a Nikon epifluorescence microscope and analyzed using YOLO v1.0 (EyeCRO, LLC, Oklahoma City, OK, USA) to identify and quantify RBPMS-positive cells. The eight individual counts were then summed and used to estimate the RGC density per mm^2^ for each flatmount. Representative TUJ1 images near the central retina were captured at 20× magnification and used to qualitatively assess axonal integrity. All imaging analyses were performed in a blinded manner. 

### 4.8. Optical Coherence Tomography

Animals were anesthetized, and their eyes were dilated according to standard procedures. Following sedation and dilation, the animals were secured on a platform and assessed using a Micron IV OCT module (Phoenix-Micron, Inc., Bend, OR, USA) for imaging. The thickness of the GCC, which consists of the retinal nerve fiber layer (RNFL), ganglion cell layer (GCL), and inner plexiform layer (IPL), was measured using peripapillary circle scans acquired from each eye, and the data were plotted as the average thickness across the scans. 

### 4.9. Optokinetic Tracking

Optokinetic tracking was performed using OptoMotry (Cerebral Mechanics Inc., Lethbridge, AB, Canada), which is designed for rodent use. For this noninvasive assessment, animals were placed on a platform surrounded by four LCD screens that resided within a light-protected box. Visual stimuli were then presented to the animal via LCD screens, and a masked observer visualized and scored the optokinetic tracking reflexes using a digital camcorder mounted on top of the box. The monitors displayed continuous vertical sine-wave gratings rotating across at 12°/s, which appeared to the animal as a virtual three-dimensional rotating sphere. The rotation of the virtual cylinder was constantly centered on the viewing position of the animal to ensure a consistent viewing distance. Tracking movements were identified as slow and steady head movements in the direction of the rotating grating. The animals were tested at spatial frequencies ranging from 0.064 to 0.514 cycles/degree to measure the spatial frequency threshold. The OptoMotry device employs a proprietary algorithm to accept input from the masked observer and automatically adjust the testing stimuli based on whether the animal exhibits a tracking reflex at a given frequency. 

### 4.10. Statistical Analysis

For comparison between the two groups, statistical analysis was performed using the F-test, followed by Student’s *t*-test or Aspin-Welch test using EXSUS software (version 10.0.7) and SAS version 9.4 (EPS Corporation, Tokyo, Japan) according to the manufacturers’ instructions. Data are presented as the mean ± standard error of the mean. *p* < 0.05 was considered statistically significant.

## 5. Conclusions

Our findings could facilitate the design of preclinical neuroprotective studies of various candidate compounds for optic nerve degeneration using BDNF as a reference agent, resulting in the refinement of drug development and furthering our understanding of the mechanisms underlying optic nerve degeneration. They may also be useful to improve the possibility of applying BDNF and its relevant pathways as a neuroprotective strategy for optic nerve injury. Future research directions should include additional investigation into the optimal dose and frequency of administration of BDNF as well as potential molecular differences between optic nerve-crushed mouse and rat eyes. Ongoing work could lead to the development of new treatment options for glaucoma and other optic neuropathies.

## Data Availability

All data generated or analyzed during this study are included in this published article.
